# An In Vitro Comparison of Marginal Fit of Single-Unit Copings Fabricated Using Computer-Aided Design/Computer-Aided Manufacturing Zirconia, Direct Metal Laser Sintering and Porcelain-Fused-to-Metal

**DOI:** 10.7759/cureus.71748

**Published:** 2024-10-17

**Authors:** Amit A Kale, Shashikala Jain, Navreet Bhasin, Sneha B Jaiswal, Ramanjeet K Grover, Balbir Singh

**Affiliations:** 1 Department of Prosthodontics, Maharaja Ganga Singh Dental College and Research Centre, Sri Ganganagar, IND

**Keywords:** cad/cam, in vitro, marginal adaptation, metal, zirconia

## Abstract

Introduction: The marginal fit of dental restorations is essential for longevity and effectiveness of fixed prostheses, particularly single-unit crowns. Direct digital scanning offers significant advantages over indirect methods, providing a non-invasive, accurate, and reproducible means to evaluate marginal fit. This study aimed to assess the marginal fit of single-unit copings fabricated using computer-aided design/computer-aided manufacturing (CAD/CAM) zirconia, direct metal laser sintering (DMLS), and porcelain-fused-to-metal (PFM) utilizing direct three-dimensional (3D) scanning.

Materials and methods: The current in vitro investigation involved 60 right mandibular second premolar typodont teeth (Dentmark, Ambala, Haryana, India) designated for single-unit fixed dental prostheses. The specimens were categorized into three groups of 20 specimens each. Direct scanning for all groups was performed using the Helios 500 intraoral scanner (Orikam Healthcare India Pvt. Ltd., Haryana, India). Upon fabrication, all copings were directly scanned using a 3D scanner (Shining 3D EX Pro, California, USA). Marginal discrepancies were assessed using the Exocad software (CAD software, Darmstadt, Germany).

Results: Mean marginal discrepancies of zirconia, DMLS, and PFM copings were evaluated and compared. Zirconia copings had the lowest mean discrepancy of 0.0172 mm whereas DMLS copings had a mean discrepancy of 0.0256 mm. The PFM copings had the highest mean discrepancy of 0.0375 mm. A one-way analysis of variance (ANOVA) indicated a significant difference in the marginal fit (p < 0.05). Post-hoc Tukey test comparisons revealed significant differences between the three groups (p < 0.05).

Conclusion: In conclusion, CAD/CAM-fabricated zirconia crowns exhibit superior marginal fit compared to DMLS and PFM copings, owing to precise digital design, milling, and material stability. DMLS copings, although less precise, still outperform PFM crowns, which display significant marginal gaps owing to the limitations of the lost-wax method.

## Introduction

The marginal fit of dental restorations is crucial for the durability and efficacy of fixed prostheses, especially single-unit crowns [1]. Poor marginal fit may lead to plaque buildup, secondary caries, periodontal issues, and early restoration failure [2]. Dental literature consistently underscores the necessity of an optimal marginal fit for the clinical success of crowns. Commonly utilized restorative materials for single-unit crowns include porcelain-fused-to-metal (PFM), zirconia, and direct metal laser sintered (DMLS) copings, each of which poses unique challenges regarding marginal precision [3,4].

The evolution of techniques for achieving an optimal marginal fit has transitioned from traditional wax patterning and metal casting to advanced computer-aided design/computer-aided manufacturing (CAD/CAM) methods [5]. These modern approaches enhance precision, minimize human error, and optimize marginal adaptation. Various in vitro and in vivo methods exist for assessing crown marginal fit. These include direct visual inspection, silicone replica techniques, and micro-computed tomography scanning [6]. Nevertheless, conventional marginal fit measurement techniques are challenging in terms of the time consumed and potential inaccuracies [7].

PFM crowns exhibit marginal accuracy limitations owing to their multi-step fabrication. Zirconia crowns produced through CAD/CAM improve precision yet present with marginal adaptation variability. DMLS copings represent a novel approach to 3D printing for metal frameworks, although accuracy issues remain a concern [8].

Direct digital scanning provides notable benefits over indirect techniques, offering a non-invasive, precise, and reproducible approach to assess marginal fit [9,10]. This technique negates the need for physical impressions and facilitates real-time evaluation, enhancing the efficiency and accuracy of crown production, thus minimizing potential errors due to the manual process. Consequently, the current investigation aimed to evaluate the marginal fit of single-unit copings produced by CAD/CAM zirconia, DMLS, and PFM through direct three-dimensional (3D) scanning.

## Materials and methods

Study design

This in vitro study was conducted at the Department of Prosthodontics at Maharaja Ganga Singh Dental College and Research Centre, Sri Ganganagar, from August 2023 to January 2024. As the study was conducted on typodont teeth, it did not require ethical committee approval as no human or human tissue was involved. 

Sample size estimation

The sample size for the study was determined using the G*Power software (Ver. 3.6.9 Heinrich-Heine-Universität Düsseldorf, Düsseldorf, Germany). A priori calculation indicated that a sample size of 60 was required to achieve 80% statistical power with 5% alpha error. The required effect size (0.42) was derived from a previous study, where the mean difference in marginal fit was 0.012 and the pooled standard deviation was 0.023 [11].

Methodology

**Figure 1:**
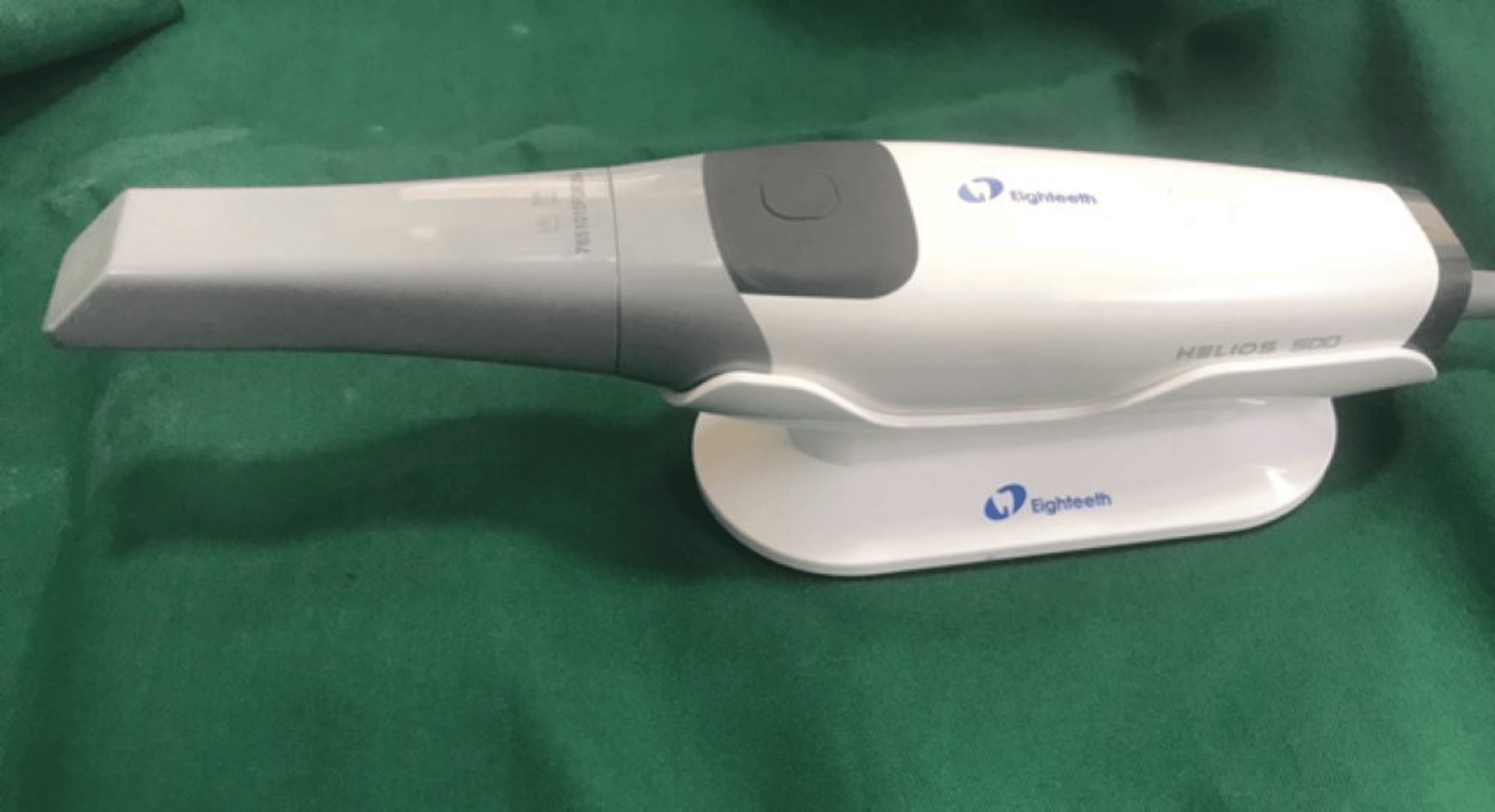
Helios 500 3D scanner Helios 500 3D scanner: Manufactured by Orikam Healthcare India Pvt. Ltd., Haryana, India.

The present study was conducted on 60 right mandibular second premolar typodont teeth (Dentmark, Ambala, Haryana, India) that were prepared to receive single-unit fixed dental prostheses. All the teeth were prepared by a single operator using standardized techniques. An occlusal reduction of 1.5 mm was performed, and a 1 mm wide shoulder margin was prepared using a shoulder diamond rotary instrument (Mani, Tamil Nadu, India). The axial preparation depth was 1 mm, whereas the occlusal depth was 1.5 mm. The digital Vernier caliper was used for measurements. For direct digitalization, an intraoral scanner was used (Helios 500, Orikam Healthcare India Pvt. Ltd., Haryana, India), and the scanning procedure was performed by the same operator, who strictly followed the manufacturer’s instructions (Figure 1). 

**Figure 2:**
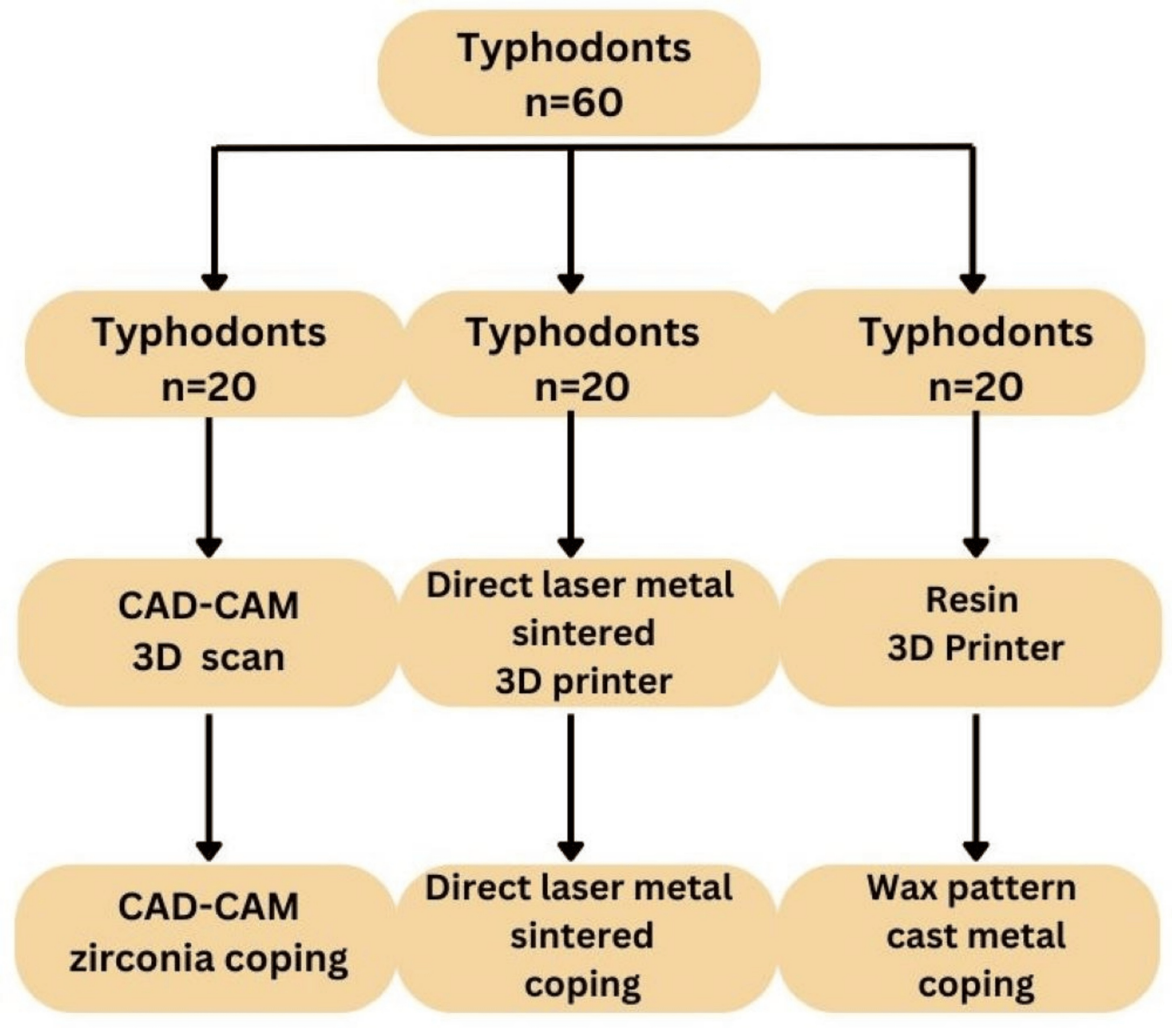
Flow chart showing group allocation CAD: Computer-aided design; CAM: Computer-aided manufacturing

The teeth were divided into three groups (20 typodonts per group). In group A, zirconia copings were designed digitally using scan data and design software (3Shape Dental System, 3Shape A/S, Copenhagen, Denmark), and fabricated using a CAD/CAM milling machine (Yenadent, Istanbul, Turkey) from zirconia blocks (Tusker Dent, Guangdong, China). In group B, digital design data were used to fabricate DMLS copings sintered from cobalt-chrome powder using a DMLS 3D printer (3D Systems, South Carolina, USA). In group C, wax copings for PFM crowns were designed using the same software and milled from wax blanks (Sigmadent, Guangdong, China) using a Resin 3D printer (Elegoo Mars 3 Pro, California, USA) (Figure 2).

**Figure 3:**
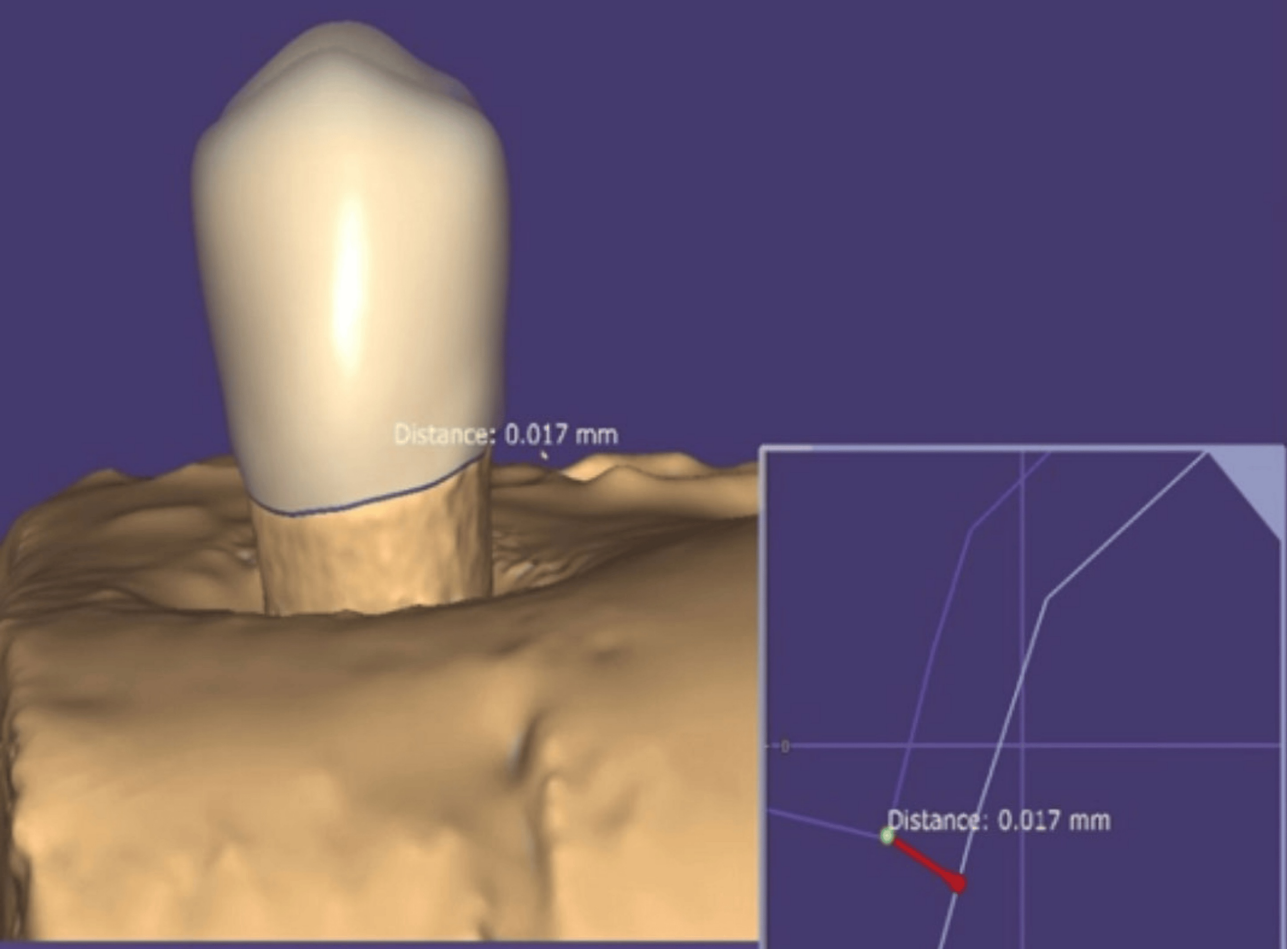
Analysis of marginal discrepancy with Exocad software Exocad software: CAD software, Darmstadt, Germany.

The wax patterns were cast into metal copings using a standard casting procedure. After fabrication, all copings were subjected to direct scanning using a 3D scanner (Shining 3D EX Pro, California, USA). Marginal discrepancies were analyzed using Exocad software (CAD software, Darmstadt, Germany) (Figure 3). All the instruments used in this study were calibrated by following the manufacturers' instructions.

Statistical analysis

The data were entered into a Microsoft Excel sheet and analyzed using IBM SPSS Statistics for Windows, Version 22 (Released 2013; IBM Corp., Armonk, New York, United States). Data normality was assessed using the Shapiro-Wilk test. Continuous variables (marginal fit measurements) were expressed as mean and standard deviation (SD) at 95% confidence interval (CI). As data were found to be normally distributed, comparisons of means between groups were performed using parametric one-way analysis of variance (ANOVA). A post-hoc analysis was conducted using the Tukey test. The level of significance was kept at p<0.05. 

## Results

The mean marginal discrepancy values for different groups were analyzed and compared. Group A demonstrated the lowest mean marginal discrepancy, followed by group B, with group C exhibiting the highest mean marginal discrepancy. A one-way ANOVA revealed a statistically significant difference in the marginal fit among the three groups (F=218.73, p=0.001). The 95% CI for the mean marginal discrepancies was 0.02 mm for group A, 0.02-0.03 mm for group B, and 0.02-0.04 mm for group C, indicating a clear variation in the accuracy of the marginal fit across the different coping materials (Table 1).

**Table 1:** Comparison of the mean marginal discrepancies (in mm) between the groups *p < 0.05: Significant, CI: Confidence interval; SD: Standard deviation; Data presented as mean ± SD.

Group	N	Mean±SD	95% CI of mean		F value	p value
Group A	20	0.0172±0.002	0.02	0.02	218.73	0.001*
Group B	20	0.0256±0.002	0.02	0.03		
Group C	20	0.0375±0.003	0.02	0.04		

Group-wise comparisons of the marginal fit revealed statistically significant differences between the three groups (group A vs. groups B and C; group B vs. group C; all p = 0.001). These findings indicate that group A provided the best marginal fit, followed by group B, with group C showing the largest marginal discrepancies (Table 2).

**Table 2:** Group-wise comparison between the groups using the Tukey test *p < 0.05: Significant, CI: Confidence interval, MD: Mean difference, SE: Standard error.

Group-wise		MD	SE	t value	p value	95% CI lower limit	95% CI upper limit
Group A	Group B	-0.01	0.001	-9.26	0.001*	-0.01	-0.01
Group A	Group C	-0.02	0.001	-20.87	0.001*	-0.02	-0.02
Group B	Group C	-0.01	0.001	-11.61	0.001*	-0.01	-0.01

## Discussion

The current study sought to assess and juxtapose the marginal fit of zirconia, DMLS, and PFM copings, all of which were produced using a direct digital scanning technique. The findings revealed that zirconia crowns exhibited the lowest mean marginal discrepancy (0.0172 mm), followed by DMLS (0.0256 mm), whereas PFM demonstrated the highest marginal discrepancy (0.0375 mm). The enhanced marginal fit of zirconia crowns can be attributed to the distinctive characteristics of zirconia as a material as well as the accuracy of the CAD/CAM technology utilized in its production [2,3]. In contrast, the copings produced through DMLS, despite demonstrating greater marginal discrepancies compared to zirconia, surpassed PFM crowns owing to the precision afforded by additive manufacturing methodologies [8,12].

The negligible marginal discrepancy noted in zirconia restorations can be explained by the application of the CAD/CAM technology, which facilitates highly accurate milling of these restorations [5]. Zirconia crowns are produced through an initial digital scanning process of tooth preparation, followed by the application of CAD software to develop a precise digital model. Subsequently, the design was milled from a pre-sintered zirconia block using the CAM technology [7]. This methodology guarantees that the marginal contours and fit of the restoration are meticulously regulated, thereby diminishing the likelihood of human error, material deformation, or inaccuracies. The intrinsic stability of zirconia throughout the sintering process further enhances its superior marginal adaptation [9]. In contrast to metal alloys, which may undergo slight distortions during casting, zirconia preserves its shape and structural integrity, leading to minimal marginal discrepancies [13,14].

Furthermore, its exceptional strength, impervious characteristics, and resistance to alterations caused by moisture or temperature fluctuations render zirconia an optimal material for the production of crowns that exhibit enhanced marginal precision [12]. Empirical research substantiates that zirconia crowns consistently demonstrate reduced marginal gaps compared to both metal-based restorations and PFM alternatives [14]. These observations are consistent with the outcomes of the present study, which revealed that zirconia exhibited minimal marginal discrepancies, underscoring its superior efficacy regarding fit and durability.

DMLS copings produced through an additive manufacturing methodology exhibited a comparatively favorable marginal fit when juxtaposed with PFM crowns. In the DMLS process, the metal alloy powder is systematically layered and subjected to sintering via a high-power laser to yield the final restorative component [3]. This methodology obviates the numerous steps characteristic of conventional casting techniques, such as wax pattern fabrication, investment, and casting, all of which are susceptible to inaccuracies and deformations. The accuracy afforded by laser sintering guarantees that the marginal contours of the DMLS coping exhibit a high degree of precision, culminating in minimal discrepancies [15]. The investigation conducted by James et al. demonstrated that DMLS copings exhibit superior marginal fit compared to traditional PFM restorations, attributable to the intrinsic precision afforded by the digital design and additive manufacturing methodology [12].

In contrast, PFM crowns are contingent on the conventional lost-wax casting technique, which encompasses several procedural steps, each with the potential for various inaccuracies. This methodology requires the fabrication of a wax pattern corresponding to the restoration, which is subsequently encased in a casting medium, followed by the actual casting of the metallic substructure [13]. The phenomena of thermal contraction and expansion during the casting phase may induce distortions that adversely affect the marginal fit. Furthermore, the application of porcelain over a metallic framework in PFM restorations adds an additional layer of complexity and potential for discrepancies [16].

Clinical implications

As zirconia crowns produced via CAD/CAM technology offer a superior marginal fit, they can lead to better long-term outcomes in terms of crown longevity and reduced risk of secondary caries or periodontal issues. In contrast, traditional PFM restorations with larger marginal gaps may pose a greater risk of clinical complications.

Limitations

The current study had several notable constraints. It was executed in an in vitro context using typodont teeth, which may not adequately simulate clinical environments, including variables such as saliva, temperature variations, and masticatory dynamics. The limited sample size and involvement of a singular operator may restrict the applicability of the results. Furthermore, only three restorative materials were analyzed, excluding other prevalent materials. The specific CAD/CAM technologies employed may not encompass the entirety of the available systems, and the investigation did not explore long-term considerations such as wear, cementation, or mechanical stress.

## Conclusions

In conclusion, zirconia crowns produced via CAD/CAM technology demonstrated superior marginal fit compared to DMLS copings and traditional PFM crowns. This enhanced marginal adaptation of zirconia is due to the accuracy of the digital design and milling, along with the stability of the material during sintering. While DMLS copings exhibited less precision than zirconia, they still benefitted from the accuracy inherent in additive manufacturing, whereas PFM restorations were characterized by significant marginal gaps owing to the limitations of the lost-wax casting method.
